# Inflammatory and Oxidative Stress Biomarkers in the Elderly, the Birjand Longitudinal Aging Study

**DOI:** 10.1155/2023/4683542

**Published:** 2023-02-21

**Authors:** Mozhgan Moshtagh, Mitra Moodi, Seyyed Ali Moezi, Farshad Sharifi, Mohammad Reza Khazdair

**Affiliations:** ^1^Social Determinants of Health Research Center, Birjand University of Medical Sciences, Birjand, Iran; ^2^Social Determinants of Health Research Center, Department of Health Promotion and Education, School of Health, Birjand University of Medical Sciences, Birjand, Iran; ^3^Cardiovascular Diseases Research Center, Birjand University of Medical Sciences, Birjand, Iran; ^4^Elderly Health Research Center, Endocrinology and Metabolism Population Sciences Institute, Tehran University of Medical Sciences, Tehran, Iran

## Abstract

Cigarette smoking is a significant risk factor for chronic and atherosclerotic vascular disease that causes preventable considerable morbidity and mortality worldwide. This study is aimed at comparing inflammation and the levels of oxidative stress biomarkers in elderly subjects. The authors recruited the participants (1281 older adults) from the Birjand Longitudinal of Aging study. They measured oxidative stress and inflammatory biomarkers serum levels in the 101 cigarettes and 1180 nonsmokers. The mean age of smokers was 69.3 ± 7.95 years, and most were male. The most percentage of male cigarette smokers have lower body mass index (BMI) (≤19 kg/m^2^). Females have higher BMI categories than males (*P* ≤ 0.001). The percentage of diseases and defects was different between cigarette and non-cigarette smoker adults (*P* ≤ 0.01 to *P* ≤ 0.001). The total white blood cells, neutrophils, and eosinophils were significantly higher in cigarettes compared to non-cigarette smokers (*P* ≤ 0.001). Besides, cigarette consumers' percentage of hemoglobin and hematocrit compared to other aged people was significantly different (*P* ≤ 0.001). However, biomarkers of oxidative stress and antioxidant levels were not significant differences between the two senior groups. Cigarette smoking in older adults was associated with increased inflammatory biomarkers and cells, but it did not find a significant difference in oxidative stress markers. Longitudinal prospective studies may help illuminate the mechanisms inducing oxidative stress and inflammation due to cigarette smoking in each gender.

## 1. Introduction

According to free radicals theory, the aging process results from cellular damages caused by metabolism, mainly mitochondrial respiration [1, 2].

Decreasing muscle mass versus increasing fat tissue can be considered chronic low-grade inflammatory conditioning in aging. It can lead to high levels of reactive oxygen species (ROS) and changing inflammatory adipocytokines and antioxidative enzymes [3, 4]. Although normal levels of ROS are essential for various cellular mechanisms [1], insufficiency of the antioxidants system and inability to detoxify the increased ROS would lead to oxidative stress (OS), or a state of imbalance between the production and regulation of ROS [5–7].

Oxidative stress in old age may harm cellular pieces of the membrane leading to loss of function and reduced metabolic efficiency [5, 6]. In this regard, OS might be the primary contributor to the formation of cardiovascular disorders owing to endothelial dysfunction through increased inflammatory and reduced anti-inflammatory cytokines [3]. Hence, inactivating nitric oxide within endothelial cells by OS may impair lipids metabolism, insulin regulation, and blood flow [3].

Genetic and social factors may harm the accumulation of OS, for example, UV light, X-ray, air pollutants, certain drugs, and tobacco smoking [1, 7]. OS is significantly an identified risk factor in most annual mortalities and fatal diseases such as cancer, cardiovascular and cerebral events due to carcinogenic, proinflammatory, and prooxidative effects [8, 9].

Smoking has been recognized as the most significant risk factor for cardiovascular disease due to widespread use in low-middle countries [10]. Existed evidence has demonstrated that tobacco smoking has a higher prevalence rate in the Asian old population, and men consume more (22.5%) compared to women (8.7%) [11]. Another study on smoking prevalence among Iranians has found cigarette consumption of nearly 13.5% for current older smoker men and 16% among past users [12].

High rates of oxidants and toxic substances in cigarettes damage airway epithelial cells, lipids, and DNA, stimulating oxidative stress, systemic inflammation, and increasing leukocytes [13]. In addition, research has found that tobacco smoking is associated with changes in inflammatory mediators, CRP, fibrinogen level, and type or number of lymphocytes identified as predictors of cardiovascular disorders [13, 14]. According to studies findings, the surge of inflammatory markers like interleukin- (IL-) 6 and C-reactive protein (CRP) is related to the number of cigarettes consumed in the life period [15, 16]. Thus, serum concentrations, accumulation, and pathophysiological effects of inflammatory markers should be higher in heavy smokers' older adults than others. Although evidence has demonstrated some atherogenic properties of tobacco smoking, probably owing to OS and inflammation, it is not clear that OS is primarily responsible for vascular dysfunction and causes inflammation or vice versa. Limitations of previous studies, including not considering the underlying characteristics of participants, persuaded researchers to perform a population-based investigation on the aged population [17, 18]. This study aimed at studying oxidative stress and inflammation changes in elderly cigarette and nonsmoker. This data can help understand the effects of cigarette consumption on the reaction of inflammatory/immune systems in old age.

## 2. Materials and Methods

### 2.1. Study Design and Participants

The aged population sample ≥ 60 years who were residents in urban and rural regions of Birjand County were invited to participate in the Birjand Longitudinal of Aging study. The participants were selected using multistage stratified cluster sampling. The city's postal areas identified 70 clusters. In each group, 20 subjects (equal numbers of males and females) were assigned [19].

Through a short interview, an expert nurse collected demographic information such as sex, age, and history of cigarette smoking. Current smokers identified with a cigarette smoking index of ≥10 pack-years (calculated by multiplying the number of cigarettes per day by the number of years spent smoking, divided by 20 cigarettes in one pack). The anthropometric measurements were also performed, and blood samples were obtained [19]. Exclusion criteria included confirmed asthma, COPD, and upper/lower respiratory tract infection in the preceding four weeks. Furthermore, the aged were completely bedridden or unable to communicate owing to severe cognitive impairment and Alzheimer's disease, and those with very short life expectancy (less than six months). The Institutional and Science committees ethically confirmed the study, and all participants gave written informed consent.

The serum concentration levels of oxidative stress and inflammatory markers from 101 cigarettes and 1180 non-cigarette smokers included as a reference control group were analyzed.

### 2.2. Serum Sample

Blood samples were drained from the cubital vein in the morning after fasting for 12 hours and collected into blood collection tubes (ORUM TADJHIZ CO, Iran, ISO 9001& 13438) without an anticoagulant using a clot separator gel. The serum was separated after 30 minutes by centrifugation at 1600g for 10 minutes, aliquoted, and stored at -80°C until use.

### 2.3. Hematologic Analysis

Hematologic indexes were analyzed by an automated hematology analyzer (SysmexKX-21 (Sysmex Corporation Kobe, Japan)). Those indexes include cell blood count (CBC), hemoglobin (Hb), hematocrit (HCT), mean corpuscular volume (MCV), mean corpuscular hemoglobin (MCH), and mean corpuscular hemoglobin concentration (MCHC).

### 2.4. Cytokine Analysis

According to the manufacturer's instructions, serum cytokine (hsCRP) profiling was performed, using Pars Azmun, Prestige 24i analyzer (Tokyo, Japan) using the serum samples without dilution. Serum cytokine levels were expressed as the median (range) in pg/ml.

### 2.5. Oxidative Stress Measurement

Evaluating oxidant stress was performed by determining the plasma concentration of markers such as porcine plasma protein hydrolysate (PPPH; radical scavenging activity (RSA) based on DPPH assay), thiol (total thiol groups based on Ellman assay), and thiobarbituric acid reactive substances (TBARS; lipid peroxidation based on thiobarbituric acid reactive substances). Furthermore, the researchers measured ferric reducing antioxidant power (FRAP) to investigate the antioxidant potential of serum levels (total antioxidant capacity (TAC) based on FRAP assay) (Table 1).

### 2.6. Ethical Approval

All the participants signed the informed consent after reading or explaining the content of the informed consent. The biobanking of the blood sample and how the researchers will be able to use it for future research were described in the consent form. The ethical research committee simultaneously approved the protocols of the current study of Endocrinology and Metabolism Research Institute (EMRI) of Tehran University of Medical Sciences (TUMS), (IR.TUMS.EMRI.REC.1396.00158) and the ethical committee of Birjand University of Medical Sciences (IR.BUMS.Rec.1397.282).

### 2.7. Statistical Analysis

Data were analyzed by the SPSS (v.22) software using the *t*-test, Mann–Whitney, and Kolmogorov-Smirnov test. Demographic and clinical data were expressed as the mean (SD), and differences between the study groups were analyzed by *t*-test (parametric data). Data for cytokine concentration levels were expressed as the median (interquartile range), and differences across the study groups were analyzed using Mann–Whitney and *t*-test. *P* < 0.05 was considered significant.

## 3. Results

A total of 1281 elderly were included in the current study. The mean age of cigarette and non-cigarette smokers was 69.3 ± 7.95 and 69.55 ± 7.49 years, respectively. Generally, the frequency of the male gender cigarette smokers was significantly more compared to the non-cigarettes smokers' group (*P* ≤ 0.001) (Table 2). The most percentage of male cigarette smokers have lower body mass index (BMI) (≤19 kg/m^2^). Also, the most percentage of females have a higher BMI (≥23 kg/m^2^) compared to male adults (*P* ≤ 0.001).

The history of diseases percentage in the total population and disease and defects history in both two groups of elderly cigarette and non-cigarette smokers are presented in Table 3. The rate of high blood pressure, diabetes, blood lipid profile, and osteoarthritis in non-cigarette smokers was significantly higher than in the smoker group (*P* ≤ 0.01 to *P* ≤ 0.001). The percentage of prostate diseases in cigarette smokers was significantly higher compared to the non-cigarette group (*P* ≤ 0.001) (Table 3). The two study groups did not differ substantially concerning inflammatory and oxidative stress biomarkers (Table 4). Total white blood cells (WBC), neutrophils (Neut), and eosinophils (Eos) in cigarette smokers were significantly higher compared to the non-cigarette smoker's group (*P* ≤ 0.01 to *P* ≤ 0.001). The lymphocyte percentage (Lymph) was considerably lower in cigarette smokers (*P* ≤ 0.05) compared to the non-cigarette smoker group (Table 5).

HCT, MCV, MCH, and MCHC were significantly higher in cigarettes than the non-cigarette smoker's group (*P* ≤ 0.05 to *P* ≤ 0.001). On the contrary, hemoglobin (Hb) was substantially lower in cigarette smokers (*P* ≤ 0.001) compared to the elderly non-cigarette smokers (Table 5).

## 4. Discussion

This investigation analyzed the inflammatory and oxidative stress biomarkers levels in non-cigarettes smokers and older adults.

The present study showed that the percentage of high blood pressure, diabetes, blood lipid profile, and osteoarthritis was significantly higher in non-cigarettes smokers than the elderly cigarette smokers. Based on the gender of participants, female is the major of non-cigarette smokers. A previous study showed that healthy females' lipid profile and blood pressure were significantly higher than healthy males [20]. Also, gender affects lipid parameters, independent of age, and menopausal status [21]. Presumably, these differences are due to the different levels of circulating sex hormones, specifically estrogens and androgens, in women versus men. It has been suggested that menopause may potentiate the age-related increase in systolic blood pressure, perhaps due to reduced arterial compliance.

The older age, male sex, diabetes, tobacco use, overweight, and obesity are independent predictors of coronary plaque and high-risk plaque in the population with a mean age of 53 years [22]. The prospective cohort study in Asia showed that secondhand smoke might raise the risk of ischemic heart disease (IHD) and the risk of incident cardiovascular disease (CVD) in middle-aged never-smoking women [23]. The study also showed that coffee consumption was associated with a low risk of all and non-Alzheimer's dementia. In contrast, smoking was associated with a high risk of non-Alzheimer's dementia in the general population with a median age of 58 years [24].

Furthermore, healthy lifestyles, including never vs. current smoking, leisure-time physical activity vs. none, and 7–9 hours vs. >9 hours of sleep, were individually associated with an 11%–25% reduced risk of Alzheimer's disease and related dementias in patients' age > 65 years old [25]. These results suggest that maintaining a healthy lifestyle is associated with a lower risk of diseases among older people.

The results of the current study show the BMI scores of female elderly were higher compared to male cigarette smokers. It has been reported that the prevalence of obesity among older *females was* higher *than among males (42.9% and 38.3*%, respectively) [26]. The prevalence of obesity among the elderly was significantly higher, and females had more predilection for obesity than males [27]. In addition, the previous study indicated that older adult females were more obese than males, and current smokers were less [28], which supports the present study's results.

The current study showed that 92% of smokers are males, and the percentage of prostate diseases was significantly higher in male cigarettes than in non-cigarette smokers. The possible relation between prostate cancer and cigarette smoking has been considered previously [29], which supported the current study results. Antioxidant parameter levels, including ferric reducing antioxidant power (FRAP), PPPH, and thiobarbituric acid reactive substances (TBARS), were decreased. Versus, the high-sensitivity C-reactive protein (hsCRP) was increased in cigarette smokers compared to the non-cigarette smoker subjects. Although these changes observed between the two groups were not significant, it may be due to the gender and small sample size of the cigarette smoker group. Based on the obtained data in the current study, BMI in female elderly was higher compared to male cigarette smokers. It has been reported that the macronutrients in the adipose tissues stimulated the release of inflammatory mediators such as TNF-*α* and IL-6 and reduced adiponectin production, predisposing to a proinflammatory state and oxidative stress [30]. Moreover, obesity was associated with an increase in the production of leptin as a proinflammatory and a reduction in adiponectin as an anti-inflammatory mediator [31]. Also, adipokines (adipocytokines) that are produced by the adipose tissue induce the production of reactive oxygen species (ROS), generating a process known as oxidative stress (OS). Obesity produces OS by mitochondrial and peroxisomal oxidation of fatty acids, which can produce ROS [32]. The results of these studies indicated that upon the increase of adipose tissue, the activity of antioxidant enzymes significantly diminished while OS and inflammatory mediators enhanced. These results indicated that although the levels of antioxidant parameters and inflammatory mediator increase in cigarette smokers, due to gender and obesity in non-cigarette smokers (female), these changes were not significant.

Exposure to tobacco promotes a more rapid decline in lung function raised oxidative stress and persistence of inflammation [33]. It has been reported that oxidative stress is generally higher in men than in premenopausal women. However, the biomarkers of oxidative stress and the risk of experiencing cardiovascular events increased in postmenopausal women [34]. Besides, different serum hydroperoxides (as the oxidative stress index) levels were observed in elderly subjects of both genders with or without coronary artery disease [35].

Our findings showed that cigarette smoking has adverse effects on hematological parameters, including white blood cell (WBC) count, Hb, HCT, MCV, MCH, and MCHC.

In our study, the number of leukocytes, hemoglobin, and hematocrit values was significantly higher in smokers than in nonsmokers. The study results revealed that the hematological parameters were significantly increased in smokers of both genders than in nonsmokers [36]. Lakshmi et al. also showed that the Hct and Hb levels were significantly higher in smokers [37], supporting the current study results. Cigarette smoking is associated with an increase in WBC count and Hb levels in total blood. Also, obesity and aging are inversely related to Hb levels in the blood [38]. An increase in hemoglobin concentration is believed to be mediated by carbon monoxide exposure, which suggests that an increase in hemoglobin level in the blood of smokers could be a compensatory mechanism.

MCV, MCH, and MCHC are the three leading red blood cell indices that help measure the red blood cells' average size and hemoglobin composition. The larger MCV, MCH, and MCHC in smokers elder were observed compared to the nonsmoker subjects, which was confirmed by other studies [39, 40].

As gender (for example, differences in body mass index) could influence inflammatory markers status, our results may be affected. The significant difference in the percentage of inflammatory markers and hematocrit of cigarette consumers follows the evidence and is in line with other research and confirms our hypothesis. Many studies have reported an association between smoking and inflammatory or immune response [41, 42]. The cross-sectional design of the current research and a more significant number of older women than males were some of its limitations. On the other hand, the majority of smokers had male gender.

## 5. Conclusion

Cigarette smoking increases hematological and inflammatory parameters associated with a greater risk for various diseases. Cigarette smoking in older adults was associated with increased inflammatory biomarkers, but any significant difference was found in biomarkers of oxidative stress. The higher BMI and obesity were associated with increased production of oxidative stress and proinflammatory markers. Longitudinal prospective studies may help determine the causal pathways and mechanisms inducing oxidative stress and inflammation due to cigarette smoking in each gender.

## Figures and Tables

**Table 1:** Oxidant stress measurement.

Test	Method	Type of kite	Company	Apparatus
PPPH	Radical scavenging activity (RSA) based on DPPH assay)	Microplate colorimetry (Zantox)	Kavosh Arian Azma Co.	Microplate reader, Biotek instruments (Epock, USA)
Thiol	Total thiol groups based on Ellman assay	Microplate colorimetry (Zantox)	Kavosh Arian Azma Co.	Microplate reader, Biotek instruments (Epock, USA)
TBARs	Lipid peroxidation based on Thiobarbituric acid reactive substances	Flourometry (Zantox)	Kavosh Arian Azma Co.	Microplate reader, Bioteck Life Science instrumentations, Citation3 imaging reader (Vermont, USA)
FRAP	Total antioxidant capacity (TAC) based on FRAP assay	Microplate colorimetry (Zantox)	Kavosh Arian Azma Co.	Microplate reader, Biotek instruments (Epock, USA)

**Table 2:** Demographic information of elderly.

Variable			Cigarette smoker	Non-cigarette smoker	*P* value
Age			69.3 ± 7.95	69.55 ± 7.49	0.691
Gender	Male		92 (16%)	474 (84%)	0.00
	Female		9 (1%)	706 (99%)	

BMI (kg/m^2^)	Male	≤19	19 (37%)	32 (63%)	0.00
		19 < 21	15 (21%)	57 (79%)	
		21 < 23	10 (10%)	86 (90%)	
		≥23	48 (10%)	415 (90%)	
	Female	≤19	0	28 (100%)	0.00
		19 < 21	1 (3%)	35 (97%)	
		21 < 23	1 (1%)	73 (99%)	
		≥23	7 (1%)	590 (99%)	

Data are expressed as *n* (%) or mean ± SD. Body mass index (BMI).

**Table 3:** Percentage of diseases and defects in elderly.

Diseases	History of diseases (*N* (%))	Cigarette smoker	Non-cigarette smoker	*P* value
High blood pressure	551 (43%)	22 (22%)	529 (48%)	0.00
Diabetes	325 (25%)	9 (9%)	316 (27%)	0.00
Heart failure	150 (12%)	11 (11%)	139 (12%)	0.790
Prostate	166 (13%)	26 (26%)	140 (12%)	0.00
Coronary angiogenesis	216 (17%)	14 (14%)	202 (17%)	0.386
Blood lipid	416 (33%)	12 (12%)	404 (34%)	0.00
Fatty liver	152 (12%)	8 (8%)	144 (12%)	0.200
Osteoarthritis	254 (20%)	9 (9%)	245 (21%)	0.004
Osteoporosis	149 (12%)	7 (7%)	142 (12%)	0.120
Visual impairment	567 (44%)	45 (45%)	522 (44%)	0.822
Uses of glasses	330 (26%)	28 (28%)	302 (25%)	0.615
Dysaudia	141 (11%)	9 (9%)	132 (11%)	0.475

Data are expressed as *n* (%).

**Table 4:** Inflammatory and oxidative stress biomarkers in elderly cigarette and non-cigarette smokers.

Variables	Cigarette smoker (mean ± SD)	Non-cigarette smoker (mean ± SD)	*P* value
hsCRP (mg/ml)	22.7 ± 24.3	15.5 ± 13.46	0.1^∗∗^
FRAP (pg/ml)	796 ± 205.8	805 ± 195.5	0.722^∗∗^
PPPH (pg/ml)	401.66 ± 97.8	421.53 ± 109.9	0.266^∗∗^
Thiol (pg/ml)	441 ± 66.15	452.7 ± 80.79	0.341^∗∗^
TBAR (pg/ml)	0.49 ± 0.1	0.47 ± 0.13	0.125^∗^

Data are expressed as mean ± SD, hsCRP: high-sensitivity C-reactive protein, FRAP: ferric reducing antioxidant power, PPPH: porcine plasma protein hydrolysate, TBARS: thiobarbituric acid reactive substances. ^∗^Mann–Whitney, ^∗∗^*t*-test.

**Table 5:** Hematological index in elderly cigarette and non-cigarette smokers.

Variables	Cigarette smoker (mean ± SD)	Non-cigarette smoker (mean ± SD)	Reference value	*P* value
WBC (Tho/*μ*l)	6.89 ± 1.77	6.2 ± 1.58	4-11	0.00^∗^

RBC (Mil/*μ*l)	5 ± 0.00	4 ± 0.00	F: 4.2-5.4	0.00^∗^
	4 ± 0.00	5.01 ± 0.00	M: 4.5-6.3	0.017^∗^

Hemoglobin (g/dL)	13 ± 0.00	13 ± 1	F: 12-16	0.00^∗^
	14 ± 1	14 ± 1	M: 14-18	0.00^∗^

Hematocrit (%)	41 ± 3	40 ± 3	F: 36-46	0.00^∗^
	42 ± 4	43 ± 3	M: 39-52	0.00^∗^

MCV (fL)	87.4 ± 6	85.7 ± 5.2	77-100	0.00^∗^

MCH (pg)	30 ± 2.59	29.2 ± 2.8	26-34	0.00^∗^

MCHC (g/dL)	34.3 ± 1.46	34 ± 1.5	30-37	0.098^∗∗^

Platelet (Tho/*μ*l)	221.59 ± 75.28	230.6 ± 66.87	150-450	0.225^∗∗^

Neutrophils (%)	54.48 ± 10.6	52.7 ± 9.63	42.2-75.2	0.095^∗∗^

Lymphocytes (%)	35.3 ± 9.8	37.85 ± 10.6	20.5-51.1	0.016^∗^

Monocyte (%)	6.9 ± 2.45	6.5 ± 2.7	1.7-9.8	0.177^∗∗^

Eosinophil (%)	3.29 ± 1.6	2.9 ± 1.4	0.0-3.0	0.013^∗∗^

Data are expressed as mean ± SD. ^∗^Mann–Whitney, ^∗∗^*t*-test. WBC: white blood cells, RBC: red blood cells, MCV: mean corpuscle (cell) volume, MCH: mean corpuscular hemoglobin, MCHC: mean corpuscular hemoglobin concentration. F: female, M: male.

## Data Availability

The datasets used and/or analyzed during the current study are available from the corresponding author on reasonable request.
